# Detection and linkage to mobile genetic elements of tetracycline resistance gene *tet*(M) in *Escherichia coli* isolates from pigs

**DOI:** 10.1186/1746-6148-10-155

**Published:** 2014-07-11

**Authors:** Sonia Jurado-Rabadán, Ricardo de la Fuente, José A Ruiz-Santa-Quiteria, José A Orden, Lisbeth E de Vries, Yvonne Agersø

**Affiliations:** 1 Departamento de Sanidad Animal, Facultad de Veterinaria, Universidad Complutense de Madrid, 28040 Madrid, Spain; 2 National Food Institute, Technical University of Denmark, Copenhagen V, Denmark

**Keywords:** *E. coli*, Pigs, *tet*(M), Horizontal gene transfer

## Abstract

**Background:**

In *Escherichia coli* the genes involved in the acquisition of tetracycline resistance are mainly *tet*(A) and *tet*(B). In addition, *tet*(M) is the most common tetracycline resistance determinant in enterococci and it is associated with conjugative transposons and plasmids. Although *tet*(M) has been identified in *E. coli*, to our knowledge, there are no previous reports studying the linkage of the *tet*(M) gene in *E. coli* to different mobile genetic elements. The aim of this study was to determine the occurrence of *tet*(A), *tet*(B), and *tet*(M) genes in doxycycline-resistant *E. coli* isolates from pigs, as well as the detection of mobile genetic elements linked to *tet*(M) in *E. coli* and its possible transfer from enterococci.

**Results:**

*tet*(A) was the most frequently detected gene (87.9%) in doxycycline-resistant isolates. *tet*(M) was found in 13.1% *E. coli* isolates. The *tet*(M) gene was detected in relation with conjugative transposons in 10 out of 36 enterococci isolates analyzed but not in any of *E. coli* isolates positive for *tet*(M). Southern blot showed that in *E. coli* and in most of the enterococci isolates the *tet*(M) gene was carried on a plasmid. According to the phylogenetic analysis, *E. coli* contained a new *tet*(M) allele grouping separately. Mating experiments revealed that *tet*(M) was carried on a mobile element successfully transferred between enterococci and between enterococci and *E. coli*.

**Conclusions:**

The detection of *tet*(M) in *E. coli* isolates from pigs was higher than expected. In our study, *tet*(M) detected in *E. coli* seems not to have been transferred from enterococci, although it can not be ruled out that the horizontal transfer of this gene occurred from other intestinal tract bacteria.

## Background

Tetracyclines, including doxycycline, are a family of antimicrobial agents that are frequently used in veterinary medicine because of their broad-spectrum of activity and their relatively low cost [1]. Besides the therapeutic use of tetracyclines, they have also been administered as growth promoters in many countries [1]. The extensive use of tetracyclines have resulted in an emergence of resistant bacteria [1]. Thus, commensal and pathogenic *Escherichia coli* isolated from pigs are often resistant to tetracycline [2-4].

Tetracycline resistance usually results from the acquisition of genes that are involved mainly in three processes: antibiotic efflux through energy-dependent membrane-associated proteins, ribosomal protection, and enzymatic inactivation of tetracycline [1,5]. More than 40 different classes of tetracycline resistance genes have been identified [5-7]. In commensal and pathogenic *E. coli*, the genes involved mainly in the acquisition of tetracycline resistance are genes encoding efflux proteins, being *tet*(A) and *tet*(B) most frequently detected [2,4,8-10]. The ribosomal protection gene *tet*(M) was first reported in *E. coli* in 2004, when Bryan *et al*. detected a *tet*(M) gene in strains from chicken and pigs that shared a 98% identity over 386 bp to a *tet*(M) gene found in *Enterococcus faecalis*[8]. Since then, this gene has also been identified in an *E. coli* from a river basin [11] and in a small number of avian, porcine, and human *E. coli* isolates [12-15].

*tet*(M) has been identified in more than 40 genera of bacteria and it has become the widest host range of any tetracycline resistance gene [5]. This may be due, at least partially, to its association with conjugative transposons [5]. In enterococci, *tet*(M) is the most common tetracycline resistance determinant and it is mainly associated with the conjugative transposon Tn*916*[16-19], although it has also been found in another conjugative transposons (Tn*5397* and Tn*5801*) and on plasmids [20-22]. To our knowledge, in *E. coli*, there are no previous reports studying the linkage of the *tet*(M) gene to different mobile genetic elements.

Enterococci and *E. coli* are natural inhabitants of the gastrointestinal tract of humans and animals. In previous studies, the *in vivo* transfer of resistance genes among intestinal tract bacteria has been showed. Thus, the transfer of resistance genes from *E. faecalis* to *E. coli* and between *E. coli* isolates in the gut has been demonstrated [23,24]. Therefore, it is possible that the finding of the *tet*(M) gene in *E. coli* strains it is due to a horizontal transfer of this gene from enterococci.

The aim of this study was to determine the occurrence of *tet*(A), *tet*(B), and *tet*(M) genes in doxycycline-resistant *E. coli* isolates from pigs, as well as the detection of mobile genetic elements linked to *tet*(M) in *E. coli* and its possible transfer from enterococci.

## Results

### Detection of tetracycline resistance genes

All of the analyzed *E. coli* isolates contained at least one of the three tetracycline resistance genes studied. The most frequently detected gene, *tet*(A), was found alone or combined with other genes in 87 of the 99 (87.9%) tetracycline-resistant isolates. *tet*(B) and *tet*(M) were detected in 42 (42.4%) and 13 (13.1%) of the *E. coli* isolates, respectively (Table 1).

**Table 1:** **Number (percentage) of tetracycline resistance genes in doxycycline-resistant *E. coli *** **isolates from pigs**

**Resistance genes**	***E. coli *** **isolates**
*tet*(A)	46 (46.5)
*tet*(B)	12 (12.1)
*tet*(A) + *tet*(B)	28 (28.3)
*tet*(A) + *tet*(M)	11 (11.1)
*tet*(A) + *tet*(B) + *tet*(M)	2 (2)

### Detection of Tn*916*-, Tn*5397*-, and Tn*5801*-like conjugative transposons

None of the 13 *tet*(M)-positive *E. coli* isolates carried the *xis*-*Tn* gene from Tn*916*, the *tndX* gene from Tn*5397*, or the *int* gene from Tn*5801*. Of the 36 *tet*(M)-positive enterococci isolates (28 *E. faecalis*, four *Enterococcus faecium*, and four *Enterococcus hirae*) selected from the pigs from which the *tet*(M)-positive *E. coli* were isolated, seven (five *E. faecalis* and two *E. faecium*) contained the *xis*-*Tn* gene*,* two (one *E. faecium* and one *E. hirae*) carried the *tndX* gene, and one (*E. faecium*) carried both the *tndX* and *int* genes. Twenty-six isolates (23 *E. faecalis* and three *E. hirae*) were negative for the *xis-Tn*, *tndX*, and *int* genes.

### Southern blot

Hybridization to the *tet*(M) probe was obtained in the plasmid DNA from the four *E. coli* isolates tested (CICYT-268, CICYT-320, CICYT-332, and CICYT-348) and from three (CICYT-381, CICYT-436, and CICYT-453) of the four enterococci isolates analyzed. The approximate size of the plasmids from the *E. coli* and enterococci isolates is around 36 Kb.

### Sequencing of the *tet*(M) gene and phylogenetic analysis

The upstream part of the *tet*(M) gene was amplified in all the *E. coli* and enterococci isolates analyzed in the study. However, the downstream part of the gene was only amplified in 12 out of the 36 enterococci isolates and it was not amplified in any *E. coli* isolate.

**Figure 1:**
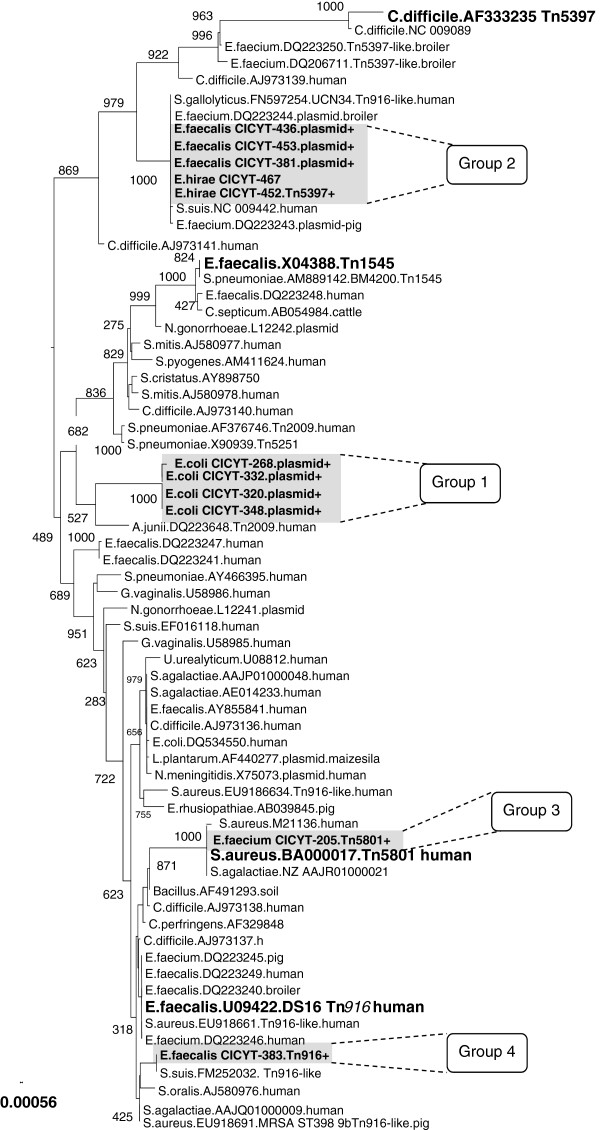
**Phylogenetic gene tree of *tet*(M).** Bootstrap values are indicated at branch points (out of 1000 generated NJ trees).

Comparison of the 11 *tet*(M) sequences selected from this study [1802 bp of the total *tet*(M) of 1920 bp] revealed five different sequence types and the pylogenetic analysis divided these into four phylogenetic groups (Figure 1). The phylogenetic tree (Figure 1) showed the plasmid-borne *tet*(M) (group 1) of the four *E. coli* isolates (CICYT-268, CICYT-320, CICYT-332, and CICYT-348) represent a new *tet*(M) allele. Four enterococci isolates negative for the *xis-Tn*, *tndX*, and *int* genes (CICYT-67, CICYT-381, CICYT-436, and CICYT-453) and one isolate positive for *tndX* from Tn*5397* (CICYT-452) contained identical *tet*(M) genes (group 2) (Figure 1). The *tet*(M) gene from enterococci isolates containing *xis*-*Tn* from Tn*916* or *int* from Tn*5801* (CICYT-383 and CICYT-205, respectively) grouped with the *tet*(M) genes of the respective transposons (Figure 1, groups 3 and 4).

### Conjugative transfer of *tet*(M) in filter mating experiments

Conjugal transfer of *tet*(M) gene between donor (three *E. faecalis* and one *E. hirae*) and recipient (*E. faecium* BM4105 and *E. faecalis* JH2-2) enterococci was observed, except from *E. hirae* to *E. faecium* BM4105, and from *E. faecalis* CICYT-381 to *E. faecalis* JH2-2 (Table 2). *tet*(M) was successfully transferred from all the four donor enterococci strains to the recipient *E. coli* CICYT70-Ri. No transfer of *tet*(M) gene was detected from *E. coli* to *E. faecium* or *E. faecalis*.

**Table 2:** Results of the mating experiments

Donor strains*	**Species**	**Transfer frequency to *E. faecalis *** **JH2-2 (tc/dn)**	**Transfer frequency to *E. faecium *** **BM4105 (tc/dn)**	**Transfer frequency to *E. coli *** **CYCIT-70-Ri (tc/dn)**
CICYT-381	*E. faecalis*	ND (<1 × 10^−9^)**	1.1 × 10^−9^	3.1 × 10^−8^
CICYT-436	*E. faecalis*	4.5 × 10^−9^	7 × 10^−9^	3.1 × 10^−8^
CICYT-453	*E. faecalis*	2.8 × 10^−8^	1.3 × 10^−8^	3.8 × 10^−8^
CICYT-467	*E. hirae*	3.6 × 10^−6^	ND (<0.7 × 10^−9^)	2.2 × 10^−8^
CICYT-268	*E. coli*	ND (<1.1 × 10^−10^)	ND (<1.7 × 10^−10^)	
CICYT-320	*E. coli*	ND (<1.5 × 10^−10^)	ND (<1.4 × 10^−10^)	
CICYT-332	*E. coli*	ND (<1.3 × 10^−10^)	ND (<1.5 × 10^−10^)	
CICYT-348	*E. coli*	ND (<2.3 × 10^−10^)	ND (<1.8 × 10^−10^)	

*tc/dn* transconjugans/donor, *ND* no transconjugants detected. *All the strains were positive for the *tet*(M) gene but negative for the Tn*916*, Tn*5397*, and Tn*5801* transposons. **(): detection limit.

## Discussion

In the present study, *tet*(A) was the tetracycline resistance gene detected most frequently, which is in agreement with a previous study carried out on *E. coli* isolated from healthy pigs [10]. On the contrary, in other studies *tet*(B) was detected more frequently than *tet*(A) in *E. coli* isolated from healthy pigs [9,25]. A negative association between the presence of *tet*(A) and *tet*(B) in *E. coli* has been described in previous studies [10,25]. It has been suggested that this negative association is probably caused by plasmid incompatibilities [26]. However, in the present study, 28 of the 99 (28.3%) *E. coli* isolates tested carried both *tet*(A) and *tet*(B) (Table 1).

The *tet*(M) gene is one of the most frequently detected tetracycline resistance determinant in enterococcal strains [16-19]. However, *tet*(M) is uncommon in Gram-negative coliforms such as *E. coli*[8,11-15]. In the present study, *tet*(M) was detected in 13 of the 99 (13.1%) tetracycline-resistant *E. coli* isolates tested. This may indicate a possible transfer of this gene from other intestinal tract bacteria, most likely from enterococci, to *E. coli*.

In enterococci *tet*(M) is often associated with conjugative transposons Tn*916*, Tn*5397*, and Tn*5801*[20,22]. Therefore, the presence of these transposons was determined in the 13 *tet*(M)-positive *E. coli* isolates and in 36 enterococci isolates selected from the pigs from which the *tet*(M)-positive *E. coli* were isolated. None of the *E. coli* isolates and only 10 of the enterococci carried some of these transposons. In contrast, Agersø *et al.*[20] detected Tn*916*-like in a high percentage (85%) of *E. faecium* isolated from pigs, although this percentage of detection was lower (53%) in *E. faecalis* strains from the same source. However, these authors [20] did not detect Tn*5397*-like among enterococci isolated from pigs, while in this work it was detected in 3 of the 36 (8.3%) isolates tested.

The absence of transposons in *E. coli* and in most of the enterococci isolates in the present study suggests that the *tet*(M) gene of these isolates is carried on a plasmid. Southern blot was performed in order to show the possible plasmid location of *tet*(M) and a positive hybridization with a *tet*(M) probe was obtained in the plasmid DNA from all the *E. coli* isolates and three of the four enterococci isolates tested.

The phylogenetic analysis shown in Figure 1 revealed a new *tet*(M) allele presents in the *E. coli* isolates which grouped separately and were only distantly related to the enterococcal *tet*(M) sequences detected in this study. Thus the origin of the plasmid-born *tet*(M) from the *E. coli* isolates is unknown, though is probably transferred from other bacteria in the intestinal tract. The *tet*(M) gene carried on a plasmid in *E. faecalis* isolates of this study was identical to *tet*(M) plasmid-borne from *E. faecium* (DQ223243) and *E. faecium* (DQ223244) isolated from pigs and broilers, respectively. In *E. faecium* CICYT-205 a Tn*5801*-like *tet*(M) gene identical to the sequence described in Tn*5801* from *Staphylococcus aureus* of human origin (BA000017) was identified. To our knowledge, this is the first report of Tn*5801*-like *tet*(M) detection in *E. faecium* and this suggests the horizontal transfer of Tn*5801* between different Gram-positive bacteria.

The mobility of *tet*(M) was investigated in filter mating experiments. The results confirmed that *tet*(M) in our enterococci isolates was linked to a mobile genetic element that could be transferred *in vitro* between enterococci, from enterococci to *E. coli*, but not from *E. coli* to enterococci. Thus, tetracycline-resistant transconjugants were obtained in all the mating experiments using *E. faecalis* as a donor and *E. faecium* BM4105 as a recipient. When *E. faecalis* JH2-2 was used as a recipient, the transfer of *tet*(M) was detected from only three of the four donor strains. Despite this result, the transfer rates for *tet*(M) between *E. faecalis* obtained in the present study were higher than those reported previously [18,19]. *tet*(M) was also transferred from *E. hirae* to the recipient strain *E. faecalis* JH2-2, but not to *E. faecium* BM4501. To the best of our knowledge, horizontal transfer of *tet*(M) from *E. hirae* to *E. faecalis* has not been reported previously.

## Conclusions

In conclusion, the detection of *tet*(M) in *E. coli* isolates from healthy pigs was higher than expected. Our results suggest that the presence of *tet*(M) in the *E. coli* isolates may be the result of the transfer of this tetracycline resistance gene from another bacteria in the intestinal tract. However, in the present study, *tet*(M) detected in *E. coli* isolates was shown to be a new allele type carried on a plasmid of unknown origin. Nevertheless, it can not be ruled out that this plasmid was transferred from other bacteria in the intestinal tract, since it is known that a gene flow between bacteria belonging to different genera occurs.

## Methods

### Doxycycline-resistant *E. coli* isolates

The doxycycline-resistant *E. coli* isolates were obtained in an ongoing research project carried out in Spain designed to evaluate the effect of the oral administration of different doses of colistin on the frequency of resistance to different antimicrobials among *E. coli* and enterococci isolates from healthy pigs. In this project, 12 healthy weaned piglets, which were obtained from the same farm and without previous exposure to antimicrobials, were examined. Animals were randomly distributed into three groups of four pigs. Groups received different doses of colistin in drinking water for 5 days. Samples of ileal content were collected at three different times. From each sample, 10 *E. coli* isolates were chosen randomly. A total of 300 *E. coli* isolates were obtained, 204 of which were doxycycline-resistant. Because of the high number of doxycycline-resistant *E. coli* isolates, a sample of 99 was randomly selected for this study.

### Detection of tetracycline resistance genes

The presence of the tetracycline resistance genes *tet*(A), *tet*(B), and *tet*(M) was determined in doxycycline-resistant *E. coli* isolates by PCR using the primers described in Table 3. The following strains were used as positive controls: *E. coli* Co228 [*tet*(A)], *E. coli* Co71 [*tet*(B)], and *E. faecalis* CG110 [*tet*(M)].

**Table 3:** Primers used in this study

**Primer use and primer**	**Sequence (5′-3′)**	**Reference**
**Detection *tet*** **(A)**		
Tet(A)-F	GCTACATCCTGCTTGCCTTC	[27]
Tet(A)-R	CATAGATCGCCGTGAAGAGG	[27]
**Detection *tet*** **(B)**		
Tet(B)-F	TTGGTTAGGGGCAAGTTTTG	[27]
Tet(B)-R	GTAATGGGCCAATAACACCG	[27]
**Detection *tet*** **(M)**		
Tet(M)-1 (266)	GTTAAATAGTGTTCTTGGAG	[16]
Tet(M)-2 (267)	CTAAGATATGGCTCTAACAA	[16]
**Detection Tn *916 *-like ( *xis *- *Tn *)**		
Tn916-1 (327)	GCCATGACCTATCTTATA	[20]
Tn916-2 (328)	CTAGATTGCGTCCAA	[20]
**Detection Tn *5397 *-like ( *tndX *)**		
Tn5397-tndX-1 (864)	ATGATGGGTTGGACAAAGA	[20]
Tn5397-tndX-2 (865)	CTTTGCTCGATAGGCTCTA	[20]
**Detection Tn *5801 *-like ( *int *)**		
intcw459-1 (1811)	CCGATATTGAGCCTATTGATGTG	[22]
intcw459-2 (1812)	GTCCATACGTTCCTAAAGTCGTC	[22]
**Amplification and sequencing *tet*** **(M)**		
TetM-upstream (526)	TTGAATGGAGGAAAATCAC	[20]
TetM-up (323)	CTGGCAAACAGGTTC	[20]
TetM sequence-1 (525)	TACTTTCCCTAAGAAAGAAAGT	[20]
TetM sequence-3 (540)	GCAGAAATCAGTAGAATTGC	[20]
TetM sequence-6 (709)	TCGAGGTCCGTCTGAAC	[22]
Reverse TetM-2 (307)	TTGTTAGAGCCATATCTTAG	[20]
TetM sequence-9 (1756)	AACAGTAAAATGTATAGAGGTG	[22]
F2R (1837)	GTGTCTTATACCATGGAAGGA	[22]
TetM-down (324)	TAGCTCATGTTGATGC	[22]
Tet(M)-1 (266)	GTTAAATAGTGTTCTTGGAG	[16]

### Detection of Tn*916*-, Tn*5397*-, and Tn*5801*-like conjugative transposons

The presence of Tn*916*-, Tn*5397*-, and Tn*5801*-like transposons was first analyzed by PCR in the *E. coli* isolates that carried the *tet*(M) gene. Later, the occurrence of these transposons was also determined in 36 *tet*(M)-positive enterococci isolates selected from the four pigs belonging to the three groups studied from which *tet*(M)-positive *E. coli* were isolated (nine isolates from each animal).

Tn*916*-, Tn*5397*-, and Tn*5801*-like were detected by amplifying the *xis-Tn*, *tndX*, and *int* genes, respectively, using the primers shown in Table 3. PCR reactions were performed as described previously [20] using *E. faecalis* CG110 (Tn*916*), *Bacillus subtilis* CU2189 (Tn*5397*), and *S. aureus* Mu50 (Tn*5801*) as positive controls.

### PCR amplification of full-length *tet*(M)

For all *E. coli* isolates and all enterococci isolates, but one, full-length *tet*(M) gene was amplified using the strategy suggested by Agersø *et al*. [20]. To amplify the upstream part of *tet*(M) by PCR, primers TetM-up (323), TetM sequence-1 (525), TetM-upstream (526), TetM sequence-3 (540), and TetM sequence-6 (709) were used (Table 3). The downstream part was amplified using the primers Reverse TetM-2 (307) and TetM sequence-9 (1756) (Table 3). One *E. faecium* isolate (CICYT-205) was suspected to contain two *tet*(M) genes. Therefore a long PCR product (4780 bp) containing the Tn*5801*-like *tet*(M) genes was amplified using primer pair TetM-upstream (526) and F2R (1837) with Phusion™ High-Fidelity DNA Polymerase (Finzymes). PCR conditions were 30 s at 98°C followed by 30 cycles of 10 s at 98°C, 30 s at 60°C and 145 s at 72°C, and a final extension for 10 min at 72°C. The sequencing primers TetM sequence-3 (540), TetM-upstream (526), TetM down (324), TetM sequence-1 (525), Tet(M)-1 (266), Reverse TetM-2 (307), and TetM sequence-9 (1756) were used (Table 3).

### Phylogenetic analysis of *tet*(M)

GenBank was searched for full length *tet*(M) genes based on the definition that *tet*(M) genes share ≥80% similarity on the amino acid level [1] and 58 nucleotide sequences were selected for the phylogenetic analysis. Eleven unique gene sequences from this study were selected to represent *E. coli* and enterococci: four of *E. coli* and four of enterococci negative for transposons [one *E. coli* and one enterococcus from each of the four animals from which *tet*(M)-positive *E. coli* were isolated] and three from enterococci positive to each of the transposons studied. A neighbor-joining (NJ) tree based on a multiple alignment of the 11 *tet*(M) sequences obtained in this study and 58 sequences from GenBank [1802 bp of the total *tet*(M) gene of 1920 bp] was constructed in Clustal X [28] and visualized by MEGA 4.0 [29]. The tree was rooted with the *tet*(O) gene (GenBank/EMBL/DDBJ accession no. Y07780) as outgroup.

### Filter mating experiments

Mating experiments were performed as described previously [30]. The conjugal transfer of *tet*(M) was analyzed in three different assays, using: enterococci as donor and recipient; *E. coli* as donor and enterococci as recipient; and enterococci as donor and *E. coli* as recipient. As donors, we selected four *tet*(M)-positive enterococci (three *E. faecalis* and one *E. hirae*) strains in which no transposons had been detected and four *E. coli* that carried the *tet*(M) gene. As recipients, *E. faecium* BM4105, *E. faecalis* JH2-2 (both resistant to rifampicin and fusidic acid), and *E. coli* CICYT70-Ri (rifampicin resistant) were used.

In the mating experiment between enterococci, transconjugants were selected on brain heart infusion (BHI) agar that contained tetracycline (8 μg/ml), rifampicin (12.5 μg/ml), and fusidic acid (12.5 μg/ml). When *E. coli* was used as a donor, transconjugants were selected in the same BHI agar, except that polymyxin B (32 μg/ml) was included instead of fusidic acid to avoid the growth of *E. coli* donors in the selection media. To select transconjugants in the mating experiment between enterococci and *E. coli*, BHI agar with rifampicin (50 μg/ml) and tetracycline (4 μg/ml) was used. Transconjugants were restricted on selective media that contained tetracycline and confirmed by the *tet*(M)-PCR screen (Table 3).

### Southern blot

Total DNA and plasmid DNA from the four *E. coli* and four enterococci used as donors in the mating experiments were purified (QIAmp DNA mini Kit and QIAGEN Tip-100, Qiagen). Southern blot was performed using the total DNA and the plasmid DNA from *E. coli* and enterococci isolates after separation by electrophoresis in 0.8% agarose gel. A specific *tet*(M) probe made from the PCR product of the TetM sequence-1 (525) and Reverse TetM-2 (307) primers was used in Southern analysis.

### Nucleotide sequence accession numbers

The sequences of the *tet*(M) gene from the *E. coli* isolates CICYT-332, CICYT-268, CICYT-320, and CICYT-348 have been deposited into GenBank under the accession numbers KJ55873-KJ55876, respectively.

## Competing interests

The authors declare that they have no competing interest.

## Authors’ contributions

SJR and YA participated in the experiments and helped to draft the manuscript. LEV participated in the experiments. RF and JARQS designed and coordinated the study and helped to draft and write the manuscript. JAO helped to draft and write the manuscript. All authors critically read and approved the final manuscript.
